# Draft Genome Sequence of Escherichia coli DSM 12242, a Highly Efficient Host Strain for the Isolation of Somatic Coliphages

**DOI:** 10.1128/MRA.00953-19

**Published:** 2019-09-19

**Authors:** Cátia Pacífico, Dmitrij Sofka, João André Carriço, Friederike Hilbert

**Affiliations:** a Department of Farm Animals and Veterinary Public Health, University of Veterinary Medicine, Vienna, Austria; b Karl Landsteiner University of Health Sciences, Krems an der Donau, Austria; c Instituto de Medicina Molecular, Faculty of Medicine, University of Lisbon, Lisbon, Portugal; Loyola University Chicago

## Abstract

Here, we report the draft genome sequence and characterization of the commercial strain Escherichia coli DSM 12242 (=ATCC 13706/60 =NZRM 3262) derived from strain C (ATCC 13706), which is suitable for the isolation of coliphages from environmental and clinical samples.

## ANNOUNCEMENT

Escherichia coli is a commensal microorganism that colonizes the gut of humans and warm-blooded animals. Strain DSM 12242 (ATCC 13706/60), a nalidixic acid-resistant derivative of strain C (ATCC 13706), allows the supplementation of this antibiotic to the medium, suppressing the background microbes present in the samples and making it a suitable indicator host for the identification of somatic coliphages (1). Derivatives of strain C, such as the better-known nalidixic acid-resistant strain WG5, are commonly used as a bacterial proxy for fecal contamination and the presence of human enteric viruses in water samples, as described in ISO method 10705-2 (2). The strain herein described was previously used as a host for the isolation of coliphages from chicken meat (3), veterinary surgery surfaces (4), and human clinical samples, such as trachea aspirates, urine, and blood (C. Pacífico, M. Hilbert, D. Sofka, N. Dinhopl, I.-J. Pap, C. Aspöck, J. A. Carriço, and F. Hilbert, unpublished data). In fact, ATCC 13706/60 was reported as giving the highest PFU count out of 16 E. coli reference culture strains tested on effluents (5), and experiments with sewage samples also showed a 3-fold higher performance for the enumeration of coliphages with ATCC 13706/60 than that with ATCC 13706 (1).

The isolate was acquired from the DMSZ and grown in modified Scholten’s agar plates for 24 to 48 h at 37°C. Genomic DNA was isolated from bacterial cells using the DNeasy blood and tissue kit (Qiagen) according to the manufacturer’s protocols. Library preparation, DNA sequencing, and sequence trimming were performed at MicrobesNG (Birmingham, United Kingdom). DNA libraries were prepared using the Nextera XT library prep kit (Illumina, San Diego, CA, USA) according to the manufacturer’s instructions, with slight modifications (2 ng of DNA was used instead of 1 ng, and the PCR elongation time was increased from 30 to 60 s). Libraries were sequenced on the Illumina HiSeq 2500 platform using a 2 × 250-bp paired-end approach. The trimmed reads were analyzed using the INNUca pipeline (6). Briefly, read quality was inspected using FastQC (http://www.bioinformatics.babraham.ac.uk/projects/fastqc/) and improved with Trimmomatic (7) version 0.38, using default parameters. Paired reads were merged with PEAR (8) version 0.9.10, using default parameters, and *de novo* assembled with SPAdes (9) version 3.13 using the “careful” option. The resulting assembly was corrected by Pilon (10) version 1.18. Whole-genome sequencing yielded a total of 1,097,039 reads, with a length between 36 and 251 bp. The genome assembly resulted in 78 contigs with an *N*_50_ value of 139,561 bp.

Strain DSM 12242 has a draft genome of 4,542,748 bp and a GC content of 50.9%. The genome coverage was calculated as 82.2×. Annotation with Prokka 1.13.3 (11) revealed the presence of 4,476 genes, 4,174 coding sequences, 11 rRNAs, 81 tRNAs, and 2 CRISPR repeats. Based on the *in silico* data, the multilocus sequence type (ST) and predicted serotype were ST 1721 (12, 13) and NT:H19 (14), respectively. ResFinder (15) identified the presence of *mdfA* (99.92% identity), a gene that encodes a multidrug resistance efflux pump. No plasmid sequences were detected using PlasmidFinder (16). The PHASTER prophage finder (17) identified two incomplete prophage regions of 8.2 kb and 16.7 kb. OrthoANI (18) revealed that this bacterium shares an average nucleotide identity of 98.99% with E. coli C (GenBank accession no. CP029371) (19) and 99.97% with strain WG5 (GenBank accession no. CP024090) (20). Nalidixic acid resistance is conferred to strain DSM 12242 by a single point mutation (Asp87→Gly) in the *gyrA* gene, while in strain WG5, it is associated with two point mutations (Asp87→Gly and Gln-106→His) (15).

### Data availability.

The genome sequence has been deposited in GenBank under the accession no. VNIO00000000, and the version described in this paper is the first version (VNIO01000000). The Illumina raw reads have been deposited in the SRA under the accession no. SRR9717097.
